# Comparative Analysis of Virulence Mechanisms of Trypanosomatids Pathogenic to Humans

**DOI:** 10.3389/fcimb.2021.669079

**Published:** 2021-04-16

**Authors:** Artur Leonel de Castro Neto, José Franco da Silveira, Renato Arruda Mortara

**Affiliations:** Departamento de Microbiologia, Imunologia e Parasitologia, Escola Paulista de Medicina, Universidade Federal de São Paulo, São Paulo, Brazil

**Keywords:** *Trypanosoma brucei*, *Leishmania* spp., *Trypanosoma cruzi*, virulence factors, immune system evasion, host-parasite interaction

## Abstract

*Trypanosoma brucei*, *Leishmania* spp., and *T. cruzi* are flagellate protozoans of the family Trypanosomatidae and the causative agents of human African trypanosomiasis, leishmaniasis, and Chagas disease, respectively. These diseases affect humans worldwide and exert a significant impact on public health. Over the course of evolution, the parasites associated with these pathologies have developed mechanisms to circumvent the immune response system throughout the infection cycle. In cases of human infection, this function is undertaken by a group of proteins and processes that allow the parasites to propagate and survive during host invasion. In *T. brucei*, antigenic variation is promoted by variant surface glycoproteins and other proteins involved in evasion from the humoral immune response, which helps the parasite sustain itself in the extracellular milieu during infection. Conversely, *Leishmania* spp. and *T*. *cruzi* possess a more complex infection cycle, with specific intracellular stages. In addition to mechanisms for evading humoral immunity, the pathogens have also developed mechanisms for facilitating their adhesion and incorporation into host cells. In this review, the different immune evasion strategies at cellular and molecular levels developed by these human-pathogenic trypanosomatids have been discussed, with a focus on the key molecules responsible for mediating the invasion and evasion mechanisms and the effects of these molecules on virulence.

## Introduction

Trypanosomatids are flagellate protozoans that can infect several types of hosts, including insects and vertebrates from different orders, in their monoxenous and dixenous forms, respectively (Nussbaum et al., 2010; Kaufer et al., 2017). Certain members of the genera *Trypanosoma* and *Leishmania* from the family Trypanosomatidae are causative agents of serious diseases in humans and have been associated with severe effects on public health globally. *Trypanosoma brucei* and *T. cruzi* cause human African trypanosomiasis (HAT) and Chagas disease, respectively. *Leishmania* parasites cause visceral and cutaneous leishmaniasis (Nussbaum et al., 2010; Kaufer et al., 2017; Mitra and Mawson, 2017).

While visceral or cutaneous leishmaniasis affects individuals worldwide (Africa, Asia, Europe, and America), Chagas disease has been primarily reported in the Americas (Central, South, and North America, and mostly in Mexico), whereas HAT has been mostly reported in African countries. Since the endemic regions may overlap, it is not uncommon to find cases of co-infection by these parasites (Mitra and Mawson, 2017; Winkler et al., 2018). The parasites are mainly dixenous and transmitted by hematophagous insects in a zoonotic or anthroponotic life cycle. Depending on the parasite species, the site of infection and development in vertebrate and invertebrate hosts will influence the severity of the disease and the virulence status (Cupolillo et al., 2000; Kaufer et al., 2017). Since these parasites must persist in insect and mammalian hosts, they develop mechanisms to infect, propagate, and survive in different environments. In insects infection, the surface proteins play a role in cell attachment and survival (Kamhawi et al., 2004; Maslov et al., 2013), whereas in mammals, the parasites produce a group of proteins and initiate processes that provide assistance during host invasion by helping the parasite evade the host immune system components (Geiger et al., 2016). Despite sharing the same evolutionary origin, *T. cruzi*, *T. brucei*, and *Leishmania* spp. adopt different methods for establishing and maintaining infection owing to the differences in the mechanisms underlying the invasion of mammalian hosts. *T. brucei* is an extracellular parasite during its life cycle (Ponte-Sucre, 2016) whereas *T. cruzi* can invade nucleated cells (Fernandes and Andrews, 2012) and *Leishmania* can only infect phagocytic cells (Walker et al., 2014). After the early invasion step, there are multiple methods which these parasites use that can modulate both innate (during the extracellular stage) and adaptive (during the intracellular stage) immune responses and induce their own growth (Zambrano-Villa et al., 2002). These include methods of host cell invasion and evasion of innate and adaptive host immune responses, which are mediated by various virulence factors, such as proteins, glycoproteins, and carbohydrates, which may be present on the cell surface or inside vesicles, or may be released by the parasite, as discussed in the subsequent sections.

This review aims to discuss the different invasion and survival mechanisms at cellular and molecular levels adopted by trypanosomatids pathogenic to humans. The components that play central roles during the invasion and evasion cycles and their influence on virulence have also been discussed.

## T. brucei

Among the trypanosomatids, *T. brucei* does not have intracellular stages in its life cycle and is exposed to constant attacks by antibody-mediated immune responses. This parasite can cause infections in humans and animals, depending on the subspecies that are inoculated by the insect vector. HAT, also known as sleeping sickness, is caused by *T. brucei gambiense* and *T. brucei rhodesiense*, whereas *T. brucei brucei* is known to cause infections in domestic and wild animals (Franco et al., 2014; Kaufer et al., 2017).

The entry of the parasites into the host is followed by the induction of innate immune responses, secretion of inflammatory cytokine and chemokines, and activation of myeloid cells for the elimination of the parasite (Ponte-Sucre, 2016; Stijlemans et al., 2016). To prevent prompt elimination, trypanosomes evade immune system attacks. One of the first lines of defense involves limiting the production of tumor necrosis factor alpha (TNF-α) by myeloid cells. This is mediated by adenylyl cyclases on the trypanosome plasma membrane, which are activated when the parasite is phagocytosed (Salmon et al., 2012; Pays et al., 2014).

The parasites encounter multiple types of host molecules associated with the humoral immune response; some of the common molecules belong to a group of high-density lipoproteins that determine the susceptibility or resistance to infection. These proteins are classified into two subsets, namely trypanolytic factors 1 and 2 (TLF1 and TLF2), which are present in human serum. There is limited information available about their levels and mode of action, besides the fact that apolipoprotein-L1 (apoL-I) is present in both complexes and is probably the primary lytic factor. In case of infection by *T. b. brucei*, apoL-I will promote parasite lysis mediated by the formation of pores in the intracellular membranes (Perez-Morga, 2005; Field et al., 2007; Wheeler, 2010; Geiger et al., 2016; Ponte-Sucre, 2016). However, the human pathogens *T. b. gambiense* and *T. b. rhodesiense* possess mechanisms that confer resistance to TLF1 and TLF2 complexes. Several processes appear to be associated with resistance in *T. b. gambiense*; these include: (1) reduction in TLF1 uptake owing to polymorphisms in its binding receptor, (2) resistance to apoL-I lysis through the expression of a truncated variant surface glycoprotein (VSG) (TgsGP), and (3) apoL-I degradation by cysteine proteases (Uzureau et al., 2013; Capewell et al., 2015). A serum resistance-associated gene encoding a truncated VSG that is actively expressed in cells resistant to human serum has been identified in the genome of *T. b. rhodesiense* (Capewell et al., 2015; Ponte-Sucre, 2016).

Once the parasites effectively counter the attack by the serum lytic system in the humoral immune response, VSG expression is another evasion mechanism used against the adaptive immune response. VSGs are highly antigenic proteins present on the surface of the parasite composed of a highly variable N-terminal domain that is exposed to the extracellular environment and a C-terminal domain that is bound to the cell membrane by a glycosylphosphatidylinositol (GPI) anchor (Ferguson et al., 1988). Before the parasites infect the human host, the expression of these proteins is activated in the salivary glands of the insect host. Following infection, VSGs help evade host responses throughout the infection cycle in the bloodstream. When these parasites are re-ingested by the tsetse fly during a blood meal, the VSGs are removed from the parasite membrane in the midgut of the insect (Pays et al., 2014; Ponte-Sucre, 2016).

Approximately 10^7^ VSGs are organized into dimers and form a protective coat on the surface of the whole cell, which shields the parasite and other membrane proteins from complement-mediated lysis by the host immune system. To elicit a protective role, the majority of the *T. brucei* population expresses the same VSG variant from a repertoire of approximately 1,000–2,000 different VSG genes (McConville et al., 2002; Geiger et al., 2016). During infection, VSGs induce an antibody response by the host immune system against the variant expressed at the moment. To reduce parasitemia, the host produces anti-VSG specific antibodies, which opsonize and lyse these parasites. However, some parasites that do not express the same variant will avoid immediate detection by the host immune system and expand their population. This mechanism confers constant antigenic variation to the parasite and allows it to sustain itself for long durations in the host bloodstream (Zambrano-Villa et al., 2002; Ponte-Sucre, 2016; Reis-Cunha et al., 2017).

Besides the major antigenic variation mechanisms described above, there are other mechanisms by which VSGs may be used by the parasite to evade the immune system of the mammalian host. Although anti-VSG antibodies can effectively eliminate the parasite, studies have shown that at a low antibody titer, the trypanosome may remove the antibodies from the cell surface through endocytosis, which occurs in the flagellar pocket, followed by the degradation of the complex in the lysosomes (McConville et al., 2002; Rudenko, 2005; Field et al., 2007; Stijlemans et al., 2016). This is possible owing to the hydrodynamic drag force resulting from directional swimming by the parasite, which pushes the VSG-bound antibodies toward the flagellar pocket and helps remove the complex *via* endocytosis (Overath and Engstler, 2004; Engstler et al., 2007; Krüger and Engstler, 2015). In addition to endocytosis in the flagellar pocket, new VSG variants are transported to the extracellular surface. Collectively, these events constitute the VSG recycling system, with constant renewal of the VSG cargo through endocytosis and exocytosis (Bangs et al., 1986; Silverman and Bangs, 2012) and a complete turnover of the entire VSG pool expressed on the cell surface within 12 min (Engstler et al., 2004).

VSGs can also be released extracellularly in a soluble form (sVSG) and eliminate bound IgGs from the surface of the parasite (Stijlemans et al., 2016). Since they are bound to the membrane by a GPI anchor, they can be cleaved upon the activation of phospholipase C (PLC), which is present in abundance in the parasite. Upon cleavage, the released VSGs carry the glycosylinositolphosphate moiety of the GPI anchor, whereas the lipid moiety (dimyristoyl glycerol) remains attached to the membrane of the parasite (Magez et al., 2002; Morrison, 2011; Geiger et al., 2016). The release of sVSG fractions during early infection has been associated with the modulation of the host immune response by the induction of a Th1 cell response and IFN-γ production. In the chronic stage of the infection, the release of these proteins have been shown to inhibit intracellular signaling and activation of macrophages (Geiger et al., 2016), elimination of preformed circulating antibodies, and formation of immune complexes (Moreno et al., 2019). Although PLC plays an important role in VSG cleavage, a previous study showed that deletions in PLC genes did not lead to a significant loss in virulence (Leal et al., 2001), which implies that antigenic variation is the primary immune escape mechanism adopted by African trypanosomes during infection (Magez et al., 2002; Morrison, 2011; Geiger et al., 2016).

VSG function is of vital importance in host-parasite interactions throughout the infection period. The variations in the sequence, expression, and release of VSGs contributes to the exhaustion of the immune system and completion of the cycle through parasite survival in the host followed by transmission back to the vector (Ponte-Sucre, 2016; Stijlemans et al., 2016).

## *Leishmania* spp

*Leishmania* spp. have evolved in environments different from those in which *T. brucei* has evolved. Infections caused by *Leishmania* spp. have widespread incidence worldwide. They are associated with a broad range of clinical and pathological manifestations (visceral and cutaneous leishmaniasis) depending on the parasite species causing the infection and the host immune response. In Africa, Asia, and Europe, *Leishmania major* is considered the primary causative agent of cutaneous leishmaniasis, and *Leishmania donovani* is mostly associated with visceral cases of the disease. In the Americas, *Leishmania braziliensis* and *Leishmania infantum* are considered the primary causative species of the cutaneous and visceral forms of the disease, respectively (Steverding, 2017; Torres-Guerrero et al., 2017).

Although this group of parasites diverged approximately 500 million years ago and evolved in different habitats worldwide (Akhoundi et al., 2016), their host-parasite interaction mechanisms appear to be conserved, with a few differences in the number and sequence of the proteins involved in this process (Reis-Cunha et al., 2017). Unlike *T. brucei*, in which the VSG variation system is the primary immune evasion tool, *Leishmania* species mediate evasion *via* various virulence factors, including proteins and carbohydrates. Glycosylinositol phospholipids (GIPLs), lipophosphoglycans (LPGs), proteophosphoglycans (PPGs), and the glycoprotein gp63 are the primary representative molecules of this group of factors that promote the invasion and maintenance of chronic infection (Franco et al., 2012; Gupta et al., 2013).

The molecules mentioned are present on the parasite membrane, and most are attached through a GPI anchor. Among these molecules, LPGs are considered the major surface glycoconjugates of the glycocalyx that cover the entire cell surface in *Leishmania*. They are present in large numbers in the promastigote form of *Leishmania*, and a significant reduction in their content is observed in the amastigote form. This molecule is composed of four distinct domains: a GPI anchor, a glycan core (carbohydrate moiety), a linear phosphoglycan chain, and a terminal neutral oligosaccharide cap. Its structure and number may vary depending on the species and stage of the parasite life cycle (Ilg et al., 1992; Ferguson, 1999; Valente et al., 2019). LPGs are one of the first targets of the immune system when the metacyclic form of *Leishmania* infects a vertebrate host. LPGs act as barriers that ﻿cause steric hindrance in the attachment of complement molecules to the surface of the parasite. This is enabled by the elongation of LPGs upon differentiation from the promastigote to the metacyclic form, which involves the doubling of linear phosphoglycan (PG) units compared to that in the promastigote (Franco et al., 2012; Gupta et al., 2013; Forestier et al., 2015). LPG also facilitates parasite attachment to and phagocytosis by macrophages, which is beneficial for *Leishmania*, since macrophages are the primary resident cells for *Leishmania*, in which the parasite replicates and differentiates during the infection process. These actions may be mediated *via* different mechanisms, such as direct binding to the macrophage receptors or through indirect interaction with proteins that contribute to the internalization of the parasite (Anversa et al., 2018). This virulence factor is associated with several other mechanisms, such as evasion of complement lysis, invasion, and survival within macrophages, as reported by Franco et al. (2012).

There is limited knowledge about the effects exerted by GIPLs on virulence, besides the fact that they are present in both cellular forms of the parasite (promastigote and amastigote) and inhibit nitric oxide (NO) synthesis by macrophages, which protects the parasite from damage (Franco et al., 2012). GIPLs have also been reported to impair the ﻿protein kinase C (PKC) pathway (Chawla and Vishwakarma, 2003). This favors parasite survival and is also associated with the activity of other virulence factors such as LPGs and gp63, although each factor adopts a different inhibitory mechanism (Gupta et al., 2013).

PPGs are mucin-like glycoproteins with a structure resembling that of LPGs; PPGs are present on the surface of *Leishmania* promastigotes and amastigotes. PPGs can also be released in abundance in the extracellular milieu by amastigotes once they move inside the parasitophorous vacuole (Ilg, 2000; Živanović et al., 2018). PPGs have been implicated in the binding of the parasite to macrophages, facilitation of parasite internalization, and modulation of the host response to the parasite. In the early stages of infection, PPGs can impair the synthesis of cytokines, such as TNF-⍺, and induce the onset of the invasion. Nonetheless, when macrophages are activated by T-cell cytokines, similar to LPGs, they stimulate the production of NO, which contributes to parasite destruction (Piani et al., 1999). PPGs have also been reported to suppress proteolytic activity in the serum and prevent opsonization of the parasite. However, PPGs also activate the complement system, which contributes to disease development (Peters et al., 1997). Collectively, PPGs help the amastigote survive in host macrophages, owing to its production at high levels in the amastigote and its extracellular release (Franco et al., 2012).

gp63 is a zinc-dependent metalloendopeptidase bound to the parasite membrane by a GPI anchor. Despite being attached to the membrane, the protein has been shown to be released in the extracellular milieu, either outside vesicles or inside them. It is considered one of the major virulence factors owing to its wide range of functions and its contribution to infection onset and maintenance (Isnard et al., 2012). Briefly, gp63 was reported to inactivate the complement cascade by inhibiting the C3b factor. This was reported to prevent the formation of the membrane attack complex (MAC) and enable the opsonization of the parasite, which facilitated its phagocytosis (Isnard et al., 2012; Shao et al., 2019). gp63 has also been shown to facilitate the binding of the parasite to macrophages through fibronectin receptors. It also exhibits protease activity by cleaving proteins from the extracellular matrix of the host, which provides *Leishmania* rapid entry into macrophages (Olivier et al., 2012).

Among other functions, gp63 affects the internal metabolism of macrophages by suppressing the production of cytokines (TNF-α and IL-12) and other cellular products (e.g., NO) that contribute in parasite elimination (Podinovskaia and Descoteaux, 2015). The inhibitory effect of this protein is also potentialized by the inactivation of mTOR kinase, which impairs the formation of an efficient translation initiation complex. Thereafter, the host macrophages become susceptible to parasite multiplication (Jaramillo et al., 2011).

During *Leishmania* infection, the virulence factors ensure parasite survival, maintenance, and proliferation inside the host, as mentioned earlier in this review. However, these factors are not restricted to immune evasion mechanisms and interactions with the immune system. There are multiple other proteins that are considered virulence factors, such as those involved in acquiring nutrients for the survival of the parasite when it is present inside the parasitophorous vacuole in macrophages (Liu and Uzonna, 2012). This acquisition is performed by parasite proteins that compete with components of host cell nutrition mechanisms, such as ﻿LIT1 and LIT2 (involved in ﻿iron sequestration) and ﻿arginase. These factors help acquire nutrients that are essential for parasite growth, and consequently, inhibit NO production by the host cell, since both processes require arginine as a basic component. ﻿Rab7 and LAMP-1 are involved in the impairment of phagosome-lysosome fusion *via* the inhibition of endosome maturation (Gupta et al., 2013).

Other proteins, such as A2, are involved in the transition of the extracellular promastigote to amastigote, which facilitates the internalization of the parasite into host cells (McCall et al., 2013). The parasite may also interact with the host cell membranes *via* amastins, a group of proteins classified into four classes (α, β, γ, and δ). The knockdown of the δ amastin gene subset was shown to impair the interaction between the parasite and the macrophage membrane, decrease amastigote viability, and attenuate the expression of the disease phenotype (de Paiva et al., 2015).

Notably, despite the fact that a parasite possesses numerous virulence factors, it is difficult to identify the most critical molecule among these, which is indispensable to the effective induction of infection by the parasite. Different studies have described knockout or knockdown experiments of the cited molecules. However, the results obtained were divergent. LPGs were shown to be critical virulence factors in *L. major* infections, but not in *L. mexicana* infections. Other studies have shown that the parasites induced an infection and were not eliminated by the immune system even in the absence of LPG or GIPL expression (Zhang and Beverley, 2010; Franco et al., 2012). In contrast, deletions in gp63 rendered the parasite significantly sensitive to complement-mediated lysis (Joshi et al., 2002; Isnard et al., 2012). A2 downregulation decreased ﻿visceralization in mouse liver cells (McCall et al., 2013), whereas amastin knockdown impaired parasite growth and attenuated the effects of the disease (de Paiva et al., 2015).

Although these results indicate that certain virulence factors may be more important for parasite survival and maintenance of infection than others, it is difficult to find a factor as important as VSG is for *T. brucei*. Possibly, in *Leishmania*, these factors take up a complementary role and work synergistically to achieve the same result. This can be easily observed in LPGs, gp63, GIPLs (39), and amastins (de Paiva et al., 2015). These are implicated in the same macrophage adhesion function; however, the molecules exhibit different modes of action and interact with different macrophage receptors. LPGs, gp63, and GIPLs also inactivate the PKC pathway during infection, which is responsible for the production of oxidative products that lead to parasite destruction (Gupta et al., 2013).

## T. cruzi

Unlike other trypanosomatids discussed in this review, *T. cruzi* is present at several cellular stages throughout the infection process, with the parasite at each stage infecting different classes of host cells using an appropriate virulence mechanism. Here, we have focused on three major mechanisms: resistance to oxidative damage, evasion of the humoral immune response, and cell invasion. Upon infection, metacyclic trypomastigotes (MTs) actively infect cells at the point of entrance (fibroblasts and surrounding tissue cells) and ﻿may be phagocytized by macrophages and dendritic cells, where they essentially need to resist oxidative stress (Osorio et al., 2012; Mesías et al., 2019).

The antioxidant mechanisms adopted by *T. cruzi* are crucial for the inactivation of reactive oxygen and nitrogen species produced by the host cells during infection initiation. To achieve this, the parasite produces several peroxidases that act on different substrates from the cellular oxidative pathway. For instance, TcGPXI and TcGPXII are glutathione peroxidases that inactivate exogenous hydroperoxides and lipid-hydroperoxides, respectively (Piacenza et al., 2013; Mesías et al., 2019); similarly, TcAPX (ascorbate-dependent heme peroxidase) disables H_2_O_2_ in conjunction with TcCPX and TcMPX, which are tryparedoxin peroxidases that also inactivate small-chain organic hydroperoxides. FeSOD (from the iron superoxide dismutase group) counters O_2_ toxicity (Piacenza et al., 2013). Proteins from the *T. cruzi* antioxidative network were found to be present at higher levels in MTs than in epimastigotes, and the expression of individual proteins such as TcCPX and TcMPX was directly correlated with cell stage differentiation (Piacenza et al., 2009).

Before escaping from the insect as an MT and after the parasite multiplies inside the host cell and egresses by disrupting the membrane as bloodstream trypomastigotes (BTs), the surface proteins of the parasite undergo extensive changes to develop mechanisms to endure complement-mediated lysis upon host infection. This evasion mechanism is mediated by the trypomastigote glycoproteins that confer resistance to the different complement pathways [classical pathway (CP), alternative pathway (AP), and lectin pathway (LP)] at these stages in the life cycle of the parasite (Osorio et al., 2012; Lidani et al., 2017).

Numerous proteins can interact with different complement pathway components. Some of these are common components of CP, AP, and LP that impair MAC formation (Cestari and Ramirez, 2010). The trypomastigote decay-accelerating factor (T-DAF) is one such component that inhibits the activation of CP and AP, and even of LP, by regulating C3 convertase expression. The *T. cruzi* complement regulatory protein (CRP), also known as gp160, blocks the same complement pathways *via* different mechanisms. T-DAF and CRP are trans-sialidase-like glycoproteins that belong to the same subfamily of the trans-sialidase superfamily of *T. cruzi* (Frasch et al., 1991; Schenkman et al., 1994). CRP is bound to the surface of the parasite through a GPI anchor and can also be released in soluble form in the extracellular milieu. Both proteins follow the same mode of action, i.e., interaction with C4b and C3b, which impairs C3b formation in CP, AP, and LP (Beucher et al., 2003; Lidani et al., 2017).

Calreticulin (TcCRT) is a calcium-binding protein that inhibits CP *via* interaction with C1q (essential for CP initiation). It also binds to a group of lectins, such as ficolins and mannose-binding lectins, which are responsible for LP activation (Ramírez-Toloza and Ferreira, 2017; Shao et al., 2019). *Trypanosoma cruzi* complement C2 receptor inhibitor tri-spanning protein (TcCRIT) impairs CP and LP activation *via* the cleavage of C2, which is a common component present in both pathways, and its interaction with C4 impairs the formation of C3 convertase (Lidani et al., 2017; Ramírez-Toloza and Ferreira, 2017; Shao et al., 2019). gp58/68 is a fibronectin/collagen receptor present on the surface of trypomastigotes that blocks AP by inhibiting the formation of C3 convertase (Fischer et al., 1988).

Following the initial host cell rupture and complement lysis evasion, the parasites enter a new cycle of infection by differentiating into extracellular amastigotes (EAs) and actively invade new host cells. With the support of adhesion and invasion molecules, the EAs attach to the surface of host cells, possibly through carbohydrate interactions (Bonfim-Melo et al., 2018). Proteins released by EAs (e.g., P21 and TcMVK) were shown to positively modulate host cell invasion. P21 reorganizes the host actin filaments in addition to inducing phagocytosis and actin polymerization (da Silva et al., 2009; Rodrigues et al., 2012). In contrast, TcMVK is attached to the host cell membrane and stimulates its uptake in HeLa cells (Ferreira et al., 2016).

Other proteins (e.g., trans-sialidases (TS), trans-sialidase-like proteins gp82 and gp85, mucins (gp35/50), and the proteases cruzipain and gp63) also exhibit virulence at different stages of the cell cycle; these proteins will be discussed in brief in this review. gp82 is one of the primary surface proteins detected in the metacyclic stage of *T. cruzi*. It is responsible for host cell adhesion and Ca^2+^ signaling cascade activation, which lead to the internalization of the parasite (Ramirez et al., 1993; Maeda et al., 2012). Experiments in mice showed that gp82 plays a major role in infection through the oral route by conferring resistance to pepsin degradation in ﻿ the gastric mucosal epithelium (Yoshida, 2009).

TS are essential to *T. cruzi* virulence (Schenkman et al., 1994; Freire-De-Lima et al., 2015). Active TS belong to group I of the TS superfamily, which includes proteins that can be linked by a GPI anchor to the cell membrane or released by the parasite upon the action of PLC on the GPI anchor, which helps modulate the host immune response (Freitas et al., 2011). These molecules can be categorized into two major groups based on the presence or absence of catalytic domains. Proteins with catalytic domains participate in the transfer of sialic acid residues (present on the surface of mammalian cells) to other cell-surface glycoconjugates in the host and to mucin-like molecules present on the membrane of the parasite. The proteins lacking the catalytic domains required for the aforementioned transfer participate in the adhesion and invasion processes by binding to host cell receptors (Schenkman et al., 1994; Freire-De-Lima et al., 2015). gp85 is another surface glycoprotein of the TS superfamily (group II) present on the surface of BTs. It contributes to cell invasion *via* a conserved FLY domain. This epitope exhibits tropism for endothelial cells, especially in the heart vessels, *via* the activation of extracellular signal-regulated kinases (Magdesian et al., 2001).

Mucins are acceptors of sialic acid residues transferred from the host donor by TS. They comprise a group of glycoconjugates present on the surface of the parasite and are classified into two groups: 1) those present only in the mammalian host (TcMUC) and providing protection against the immune system; 2) those that protect the parasite in the insect vector (TcSMUG). As protective molecules, mucins in the mammalian host receive sialic acid from TS, which is used in adhesion, immunomodulation of host defense, and complement evasion (Almeida et al., 1999; Acosta-Serrano et al., 2001). In insect vectors, mucins protect the parasite from digestive proteases, besides contributing to adhesion events (Herreros-Cabello et al., 2020).

Mucin-associated surface proteins (MASPs), which exhibit structural similarities with TcMUC, constitute a large group of proteins primarily found in the parasites in infectious stages (MTs and BTs); these proteins contribute to invasion by facilitating endocytosis (dos Santos et al., 2012), and also contribute to the survival and multiplication of intracellular amastigotes (Bartholomeu et al., 2009). gp35/50 is a mucin-like protein complex that is also present in MTs; it plays a role considerably similar to that of gp82 and promotes the internalization of MTs in the host cell using Ca^2+^ (Maeda et al., 2012).

In addition to these proteins, cysteine endopeptidases have also been implicated in the virulence of several parasites. In *T. cruzi*, cruzipain is the best-characterized protein from this group. It is expressed in the major stages of the parasite life cycle and is primarily present in lysosome-associated organelles, besides being present on the membrane in amastigotes (Alvarez et al., 2012). Cruzipain contributes to the proteolytic degradation of host tissues, host cell invasion, and immune evasion, possibly through the degradation of human IgG moieties (McKerrow et al., 2006; Osorio et al., 2012). Several other cell invasion-associated proteins have been identified, and their functions have been discussed in detail in earlier publications (Osorio et al., 2012; Leiria Campo et al., 2016; Horta et al., 2020).

Similar to that in *Leishmania*, gp63 proteases are also present in *T. cruzi*. The genes encoding these proteins undergo expansion, similar to the genes encoding TS and TS-like glycoproteins, mucins, and MASPs; this suggests that the proteins may be involved in host immune system evasion by the parasite (Garcia et al., 2007). There are two types of gp63 proteases: gp63-I and gp63-II. gp63-I is known for its metalloprotease activity and membrane attachment *via* GPI anchoring, and is detected in the three cellular stages of the parasite. Certain proteins in the gp63-I group are glycosylated, whereas others are non-glycosylated. The glycosylated forms are present on the membrane of the amastigote and epimastigote, whereas the non-glycosylated forms are present intracellularly, located close to the kinetoplast and flagellum pocket in the metacyclic trypomastigote. Compared to what is known about gp63-II proteins, there is limited information available on the functions and location of gp63-I proteins, besides the fact that they do not contain the GPI anchor domain sequence (Pech-Canul et al., 2017).

## Concluding Remarks

**Figure 1:**
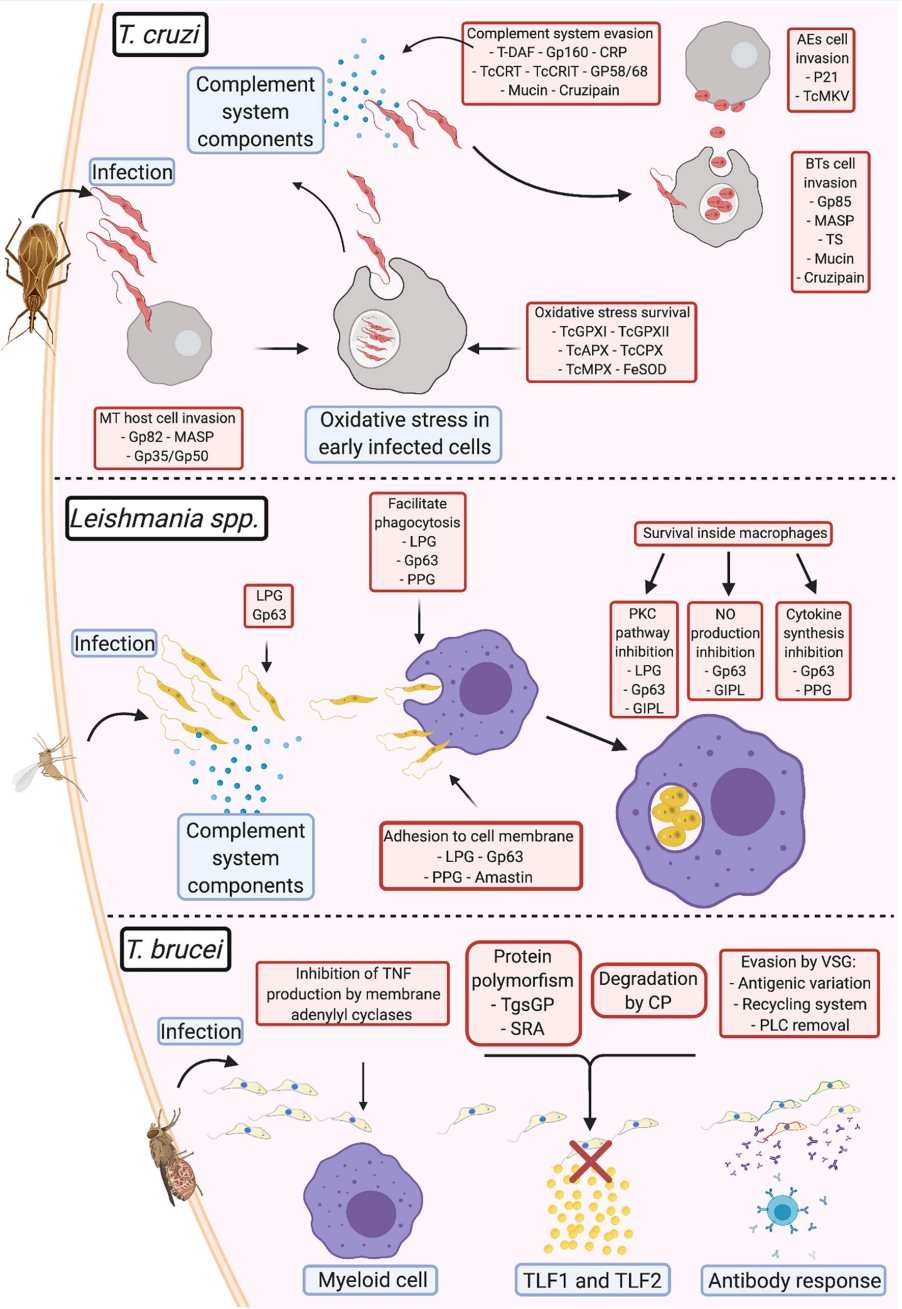
Comparison of virulence strategies adopted by human pathogenic trypanosomatids ***Trypanosoma cruzi***: During infection, the metacyclic trypomastigotes invade cells at the site of entrance aided by gp82, MASP, and gp35/gp50; once inside the cell, TcGPXI TcGPXII, TcAPX, TcCPX, TcMPX, and FeSOD protect the parasite from the oxidative stress response; after multiplication and return to the extracellular milieu, *T. cruzi* evades the complement system (using T-DAF, gp160, CRP, TcCRT, TcCRIT, gp58/68, mucin, and cruzipain) until they invade new cells *via* membrane adhesion (using gp85, MASP, TS, mucin, and cruzipain). After a fresh round of multiplication and egression from the infected cell, if the parasite exits as an extracellular amastigote, it can directly infect new cells using surface protein adhesion (P21 and TcMKV). ***Leishmania* spp.**: Complement system evasion in *Leishmania* is primarily mediated by gp63 and lipophosphoglycans (LPGs). This interaction opsonizes the parasites and facilitates adhesion to the membrane of phagocytic cells, with assistance from proteophosphoglycans (PPGs) and amastins. Upon entry into the parasitophorous vacuole, gp63, LPGs, glycosylinositol phospholipids, and PPGs block the pathways that produce components that damage *Leishmania* and promote parasite survival and multiplication. *T. brucei*: To prevent immediate elimination, the parasite suppresses TNF-α production from the membrane adenylyl cyclases in myeloid cells. Evasion from TLF1 and TLF2 by *T. b. gambiense* is mediated *via* cysteine proteases and polymorphisms in the TgsGP receptor; *T. b. rhodesiense* prevents interaction with the same lytic factors using the serum resistance-associated proteins present on the membrane. In *T. brucei*, variant surface glycoproteins (VSGs) are primarily responsible for antibody response evasion. This involves: (1) prevention of the binding of antibodies to the cell membrane *via* antigenic variation, (2) elimination of antibodies bound to VSG by endocytosis of the VSG-antibody complex in the VSG recycling system, or (3) cleavage of the antigen-antibody complex by phospholipase C (PLC).

Despite sharing a close evolutionary relationship and exhibiting similarities in terms of morphology and genetic composition, the different clinical presentations of the diseases caused by *Leishmania* and trypanosomes and the differences between the host environments clearly indicate the existence of specific virulence mechanisms in the different parasites. Since *T. brucei* is predominantly extracellular, its mechanisms are mostly focused on escaping the humoral immune response and neutralizing the lytic effects of the complement and antibody attacks. These actions (escape from the complement and lytic systems) are well conserved among the three trypanosomatids, despite being mediated by different sets of proteins, as shown in **Figure 1**. Unlike *T. brucei*, both *Leishmania* and *T. cruzi* have intracellular stages in their life cycles, which are associated with complex mechanisms typical to the particular infection stage; however, there are a few important differences between the intracellular stages in the two parasites. *Leishmania* amastigotes are generally present inside macrophages and immune system cells that phagocytose the parasite; therefore, they tend to induce their own opsonization and bind to immune cell receptors to stimulate and facilitate their incorporation into these cells. These actions are also performed by *T. cruzi* during the initial stages of infection. However, through evolution, *T. cruzi* has acquired methods to actively infect host cells even when it is absent from the immune system, which confers the ability to avoid detection even in cases of acute infection and results in the detection of the parasite years after the infection, when the health of the host organs is significantly compromised.

Notably, the virulence mechanisms described in the review and illustrated on **Figure 1** (e.g., complement system evasion, adhesion, and host cell invasion) are mediated by a group of different parasite molecules that eventually achieve the same result. Therefore, it is difficult to determine the proteins responsible for the progression of each evasion process, since host-parasite interactions appear to occur stochastically, depending on the strain of the parasite, the virulence factor, and the availability of its receptor during infection. In case a virulence factor is blocked or unavailable, others that mediate the same function would play the role, perhaps with different efficiencies. However, further studies are required to confirm this. This observation also draws attention to the potential of virulence factors as targets for drug-based therapy, immunotherapy, or vaccine development. Since most studies in this area of research only tend to focus on single targets, studies on the identification of multiple targets may help improve the efficiency in preventing infection.

## Author Contributions

AC wrote the manuscript and prepared the figure. JS and RM reviewed and edited the manuscript and figure. All authors contributed to the article and approved the submitted version.

## Conflict of Interest

The authors declare that the research was conducted in the absence of any commercial or financial relationships that could be construed as a potential conflict of interest.
